# Lipopolysaccharide (LPS)-induced autophagy is involved in the restriction of *Escherichia coli* in peritoneal mesothelial cells

**DOI:** 10.1186/1471-2180-13-255

**Published:** 2013-11-13

**Authors:** Juan Wang, Xiaoran Feng, Youjia Zeng, Jinjin Fan, Juan Wu, Zhijian Li, Xinhui Liu, Rong Huang, Fengxian Huang, Xueqing Yu, Xiao Yang

**Affiliations:** 1 Department of Nephrology, The First Affiliated Hospital, Sun Yat-sen University, 58th, Zhongshan Road II, Guangzhou 510080, China

**Keywords:** Autophagy, *Escherichia coli*, Cell defense, Lipopolysaccharide (LPS), Toll-like receptors (TLR), Peritoneal mesothelial cell

## Abstract

**Background:**

Host cell autophagy is implicated in the control of intracellular pathogen. *Escherichia coli* (*E.coli*) is the most common organism caused single-germ enterobacterial peritonitis during peritoneal dialysis. In this study, we investigated autophagy of peritoneal mesothelial cells and its role in defense against *E.coli.*

**Results:**

Autophagy in human peritoneal mesothelial cell line (HMrSV5) was induced by lipopolysaccharide (LPS) in a dose-dependent and time-dependent way, which was demonstrated by increased expression of Beclin-1 and light chain 3 (LC3)-II, the accumulation of punctate green fluorescent protein-LC3, and a higher number of monodansylcadaverine-labeled autophagic vacuoles. After incubation of HMrSV5 cells with *E.coli* following LPS stimulation, both the intracellular bactericidal activity and the co-localization of *E.coli* (K12-strain) with autophagosomes were enhanced. Conversely, blockade of autophagy with 3-methyladenine, wortmannin or Beclin-1 small-interfering RNA (siRNA) led to a significant reduction in autophagy-associated protein expression, attenuation of intracellular bactericidal activity, and reduced co-localization of *E.coli* with monodansylcadaverine-labeled autophagosomes. In addition, treatment of HMrSV5 cells with LPS caused a dose-dependent and time-dependent increase in Toll-like receptor 4 (TLR4) expression. Both knockdown of TLR4 with siRNA and pharmacological inhibition of TLR4 with Polymyxin B significantly decreased LPS-induced autophagy. Furthermore, TLR4 siRNA attenuated remarkably LPS-induced intracellular bactericidal activity.

**Conclusions:**

Our findings demonstrated for the first time that LPS-induced autophagy in peritoneal mesothelial cells could enhance the intracellular bactericidal activity and the co-localization of *E.coli* with autophagosomes. The activation of TLR4 signaling was involved in this process. These results indicate that LPS-induced autophagy may be a cell-autonomous defense mechanism triggered in peritoneal mesothelial cells in response to *E.coli* infection.

## Background

Autophagy is a conserved proteolytic mechanism by which cytoplasmic components, including damaged organelles, toxic protein aggregates and intracellular bacteria and viral pathogens are sequestered in a specialized double-membrane-bound autophagosome and delivered to the lysosome for bulk degradation and subsequent recycling [1-3]. It was well known that autophagy plays an important role not only in cell homeostasis, but also in innate immunity [3-7]. Invading bacteria could be driven to the autophagosome–lysosome pathway for degradation (‘xenophagy’) which protects the host against pathogen colonization [8,9]. It has been reported that autophagy is necessary for cells to restrict many pathogens such as *Mycobacterium tuberculosis*[7,10], Group A *Streptococcus*[5], *Salmonella enterica*[6], *Francisella tularensis*[1] and *Rickettsia conorii*[1].

Peritoneal dialysis (PD)-related peritonitis represents a serious complication and is the most important cause leading to the dropout in PD patients [11]. *Escherichia coli* (*E.coli*) is the most common organism caused single-germ enterobacterial peritonitis during PD [12,13]. It was noticed in recent years that a change in the virulence of *E. coli* peritonitis episodes resulted in high rates of treatment failures and even mortality [12,13]. Lipopolysaccharide (LPS) is the biologically active constituent of endotoxins derived from the cell wall of Gram-negative bacteria [10,14], which is a potent inducer of autophagy in many cell lines, including macrophages [10], human keratinocytes [15], and myoblasts [16]. However, the induction of autophagy by LPS in peritoneal mesothelial cells (PMCs), which provides a nonadhesive and protective layer in the abdominal cavity against the invasion of foreign particles and injury [17], and the role of autophagy in the elimination of *E. coli* from PMCs have not been studied yet. The objective of present study was to investigate the autophagy induced by LPS in PMCs and its role in defense against *E. coli.* We were specifically interested in determining whether autophagy contributes to *E.coli* survival or death.

## Methods

### Materials

Dulbecco’s modified Eagle’s medium/F12 (DMEM/F12) and fetal bovine serum (FBS) were purchased from Gibco BRL (Grand Island, NY, USA). Ultra-pure LPS (upLPS) from *Escherichia coli* (O111:B4) was obtained from Invivogen (San Diego, CA, USA). Anti-LC3, anti-TLR4 and anti-Beclin-1 were from Abcam (Cambridge, UK). Vimentin was from Boster Biological Technology (Wuhan, China). Secondary antibodies were from Cell Signaling Technology (Danvers, MA, USA). Anti-cytokeratin 18 (CK-18), 3-methyladenine (3-MA), wortmannin (Wm), monodansylcadaverine (MDC), 3-[4, 5- dimethylthiazol −2 -yl]-2, 5-diphenyltetrazolium bromide (MTT), 4’,6-Diamidino-2-phenylindole dihydrochloride (DAPI), Polymyxin B (PMB) and gentamicin were from Sigma-Aldrich Co.. Fluorescent *E.coli* (K-12 strain) BioParticles, Lipofectamine 2000 and Annexin V-FTIC Apoptosis Detection Kit were from Invitrogen Life Technologies (Carlsbad, CA, USA). The green fluorescent protein (GFP)-LC3 fusion plasmid was kindly provided by Professor Xiaofeng Zhu. Beclin-1 specific small-interfering RNA (siRNA) and TLR4 specific siRNA was from Shanghai GenePharma Co., Ltd. (Shanghai, China).

### Cell culture and viability studies

The simian virus 40 (SV40)-immortalized human peritoneal mesothelial cell line (HMrSV5) has been described previously [17,18]. HMrSV5 cells were cultured in DMEM/F12 medium containing 10% FBS in a humidified atmosphere consisting of 95% O_2_ and 5% CO_2_ at 37°C. The cell line was identified by phase contrast microscopy and immunofluorescence analysis. The effect of LPS on the viability of cultured HMrSV5 cells was determined by MTT assay [17,19] and flow cytometric analysis [20].

### Immunofluorescence co-staining of CK-18 and vimentin

After fixed in 4% paraformaldehyde for 15 min at room temperature, cells were permeabilized with 0.1% Triton X-100, followed by incubating with 5% BSA in PBS for 60 min at room temperature to block nonspecific binding. Then cells were stained with mouse anti-vimentin and mouse anti-cytokeratin 18 in PBS containing 5% BSA at 4°C overnight. Cells were incubated with secondary antibody for 1 hour at room temperature. Finally, coverslips were sealed with mounting medium. Images were collected by an LSM 510 confocal immunofluorescence microscope (Carl Zeiss, Inc., Jena, Germany).

### Measurement of autophagy by immunoblotting

Equal amounts of protein were separated on 15% SDS-polyacrylamide gels and transferred to polyvinylidene difluoride (PVDF) membranes. After blocking with 5% nonfat dry milk in Tris-buffered saline for 60 min at room temperature, the membranes were incubated at 4°C overnight with primary antibody. Following incubation with secondary antibodies, the protein bands were detected by an enhanced chemiluminescence system. Densitometric quantification of band intensities was determined using an image analysis program (FluorChem 8900; Alpha Innotech Corp, San Leandro, CA, USA).

### Transfection of HMrSV5 cells with GFP-LC3 plasmid

HMrSV5 cells at 50-70% confluence were transiently transfected with 2 μg/ml GFP-LC3 plasmid DNA per dish which was performed with Lipofectamine 2000. After treatments as shown in the figure legends, the cells were fixed with 4% paraformaldehyde and nuclei were labeled with DAPI. Autophagy was assessed by the formation of fluorescent autophagosome puncta. Cells with more than 10 puncta indicated the GFP-LC3 positive cells. Values were calculated from 100 cells/sample.

### Detection of autophagic vacuoles by MDC

Treated cells were washed 3 times with PBS and then incubated with 0.075 mM MDC in DMEM/F12 at 37°C for 10 min. The cells were then immediately observed under a fluorescence confocal microscope equipped with the appropriate filters, where MDC exhibits autofluorescence at wavelengths of 365 and 525 nm for excitation and emission, respectively.

### SiRNA gene silencing of Beclin-1 or TLR4

Knock down of Beclin-1 or TLR4 in HMrSV5 cells was obtained by utilizing complementary sense and antisense oligonucleotides to human Beclin-1 or TLR4 (Beclin-1 siRNA: sense, 5′-CUCAGGAGAGGAGCCAUUUTT-3′, antisense, 5′-AAAUGGCUCCUCUCCUGAGTT-3′; TLR4 siRNA: sense, 5′-CCACCUCUCUACCUUAAUATT-3′, antisense, 5′-UAUUAAGGUAGAGAGGUGGTT-3′). A non-targeting siRNA pool was applied as a control (negative control siRNA for Beclin-1 siRNA: sense, 5′-UUUAGCCGAUACUGCCUAGTT-3′, antisense, 5′-CUAGGCAGUAUCGGCUAAATT-3′; negative control siRNA for TLR4 siRNA: sense, 5′-UUCUCCGAACGUGUCACGUTT -3′, antisense, 5′-ACGUGACACGUUCGGAGAATT-3′). HMrSV5 cells were transfected with 1 μg of each duplex using Lipofectamine 2000.

### Bacterial killing assay

The *E. coli* strain (ATCC: 25922) was resuspended in saline without antibiotics prior to infection of HMrSV5 cells. HMrSV5 cells were plated at a density of 5.0 × 10^5^ cells per well and then treated as shown in the figure legends. *E.coli* was added at a MOI of 20 and incubated at 37°C for 1 hour (t = 0). Then, HMrSV5 cells were washed with cold PBS to remove non-adherent bacteria and stop additional bacterial uptake. Meanwhile, gentamicin (10 μg/ml) was added to limit the growth of extracellular bacteria. The cells were lysed at further 30 min, 60 min and 90 min respectively (t = 30, 60, 90) with sterile distilled water. The number of viable bacteria (colony forming units, c.f.u.) released from cells was detected by plating serial dilutions of bacteria on Luria Bertani (LB) agar plates. Bactericidal activity was analyzed by the percentage of remaining *E.coli* (%) which was was calculated as (remaining bacteria at each time point/bacteria present at 0 min) × 100.

### Analysis of *E. coli* co-localization with autophagosomes by immunofluorescence

Cells were infected with *E. coli* (K-12 strain) BioParticles at a MOI of 20:1 for 1 hour. Following phagocytosis, cells were treated as shown in the figure legends. Subsequently, the cells were washed 3 times with PBS and incubated with 0.075 mM MDC in DMEM/F12 at 37°C for 10 min. The cells were observed under a fluorescence confocal microscope equipped with the appropriate filters where MDC exhibits autofluorescence at wavelengths of 365 and 525 nm for excitation and emission, respectively.

### Transmission electron microscopy

Cells were fixed at room temperature with former fixative (0.1 mol/l PBS containing 2.5% glutaraldehyde, and 2% paraformaldehyde). The samples were postfixed with 1% osmium tetroxide, subsequently incubated with 1% uranyl acetate, then dehydrated through increasing concentrations of ethanol, and gradually infiltrated in LX-112 medium. Thin sections of each sample were stained with 2% uranyl acetate and lead citrate, and then analyzed under a JEM 1010 transmission electron microscope (JEOL, USA, Inc., Peabody, MA).

### Statistical analysis

Quantitative data were expressed as means ± standard deviations. The statistical differences in multiple groups were determined by one-way ANOVA followed by Student–Neuman–Keuls test. Statistical differences between two groups were analyzed by two-tailed unpaired Student’s *t*-test. All calculations were performed using SPSS 13.0 statistical software (Armonk, NY, USA). A value of *p* < 0.05 was considered significant.

## Results

### Characterization of human peritoneal mesothelial cell line (HMrSV5) in culture

**Figure 1:**
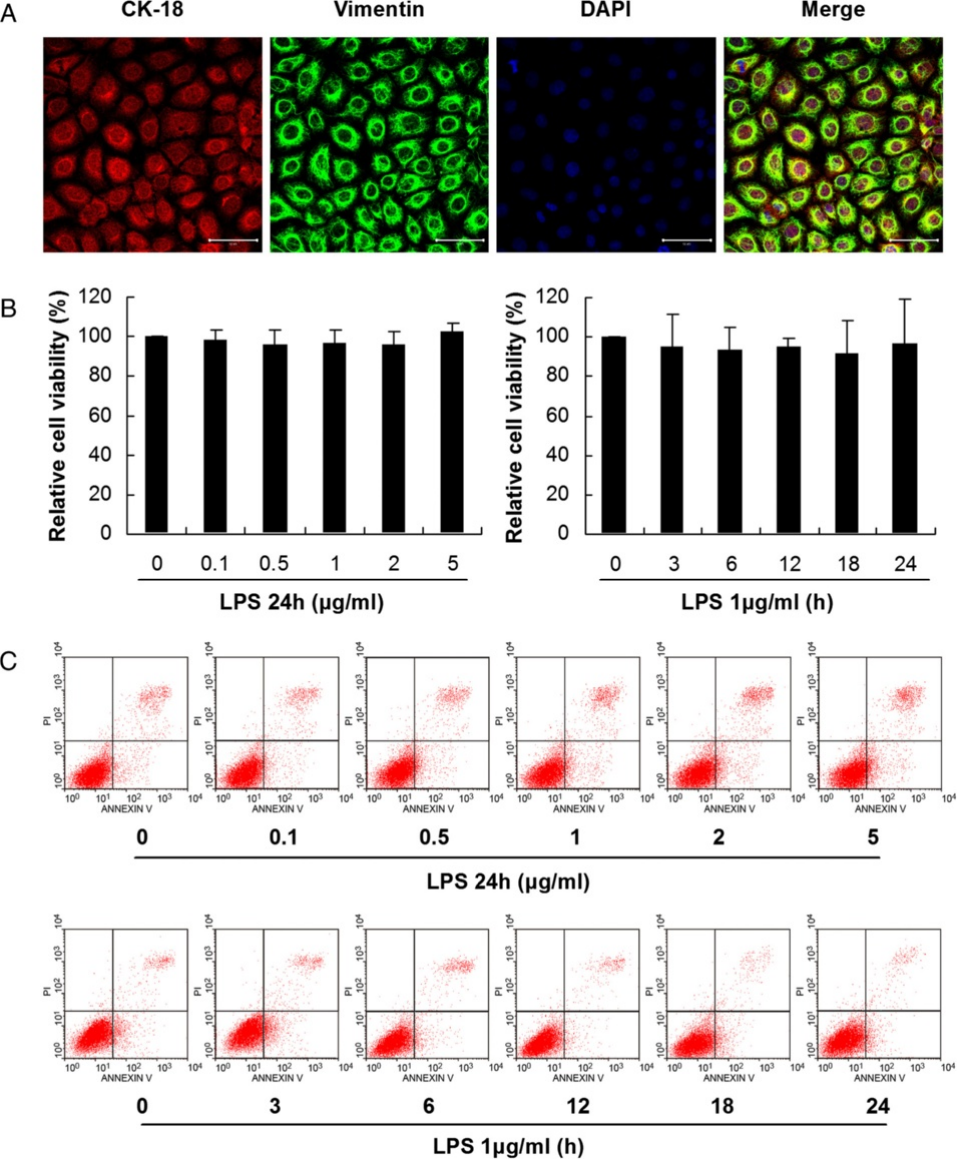
**Characterization and analysis of cell viability in HMrSV5 cells. (A)** Confluent PMCs were positive for cytokeratin 18 and vimentin. Scale bars: 50 μm. **(B)** Cell viability was determined by MTT assay. The left panel shows the viability of HMrSV5 cells exposed to different concentrations (0, 0.1, 0.5, 1.0, 2.0 and 5.0 μg/ml) of LPS for 24 hours. The right panel shows the viability of HMrSV5 cells exposed to 1 μg/ml LPS for different times (0, 3, 6, 12, 18 and 24 hours). The results are presented as a percentage of the MTT absorbance of untreated cells (100%). Data represent mean values ± SD (n ≥ 3). **(C)** Detection of cell viability by flow cytometric analysis. The upper panel shows dose responses for LPS-induced apoptosis over 24 hours in HMrSV5 cells. The lower panel shows apoptosis in cells treated with 1.0 μg/ml LPS for 0, 3, 6, 12, 18 and 24 hours.

Confluent HMrSV5 cells exhibited multipolar with a uniform cobblestone-like appearance under the phase contrast microscope. Immunofluorescence analysis showed positive staining for cytokeratin 18 and vimentin (Figure 1A), but negative staining for factor VIII associated antigen and CD45 (data not shown).

### Effects of LPS on cell viability

Following exposure of HMrSV5 cells to 1.0 μg/ml LPS for 0, 3, 6, 12, 18 and 24 hours, or to the concentrations of 0, 0.1, 0.5, 1.0, 2.0 and 5.0 μg/ml LPS for 24 hours, MTT assay showed no significant changes in cell viability (Figure 1B). Flow cytometric analysis also indicated that the rates of apoptosis in HMrSV5 cells did not change statistically after treatments of LPS as described above (Figure 1C).

### Autophagy in HMrSV5 cells was induced in response to LPS stimulation

**Figure 2:**
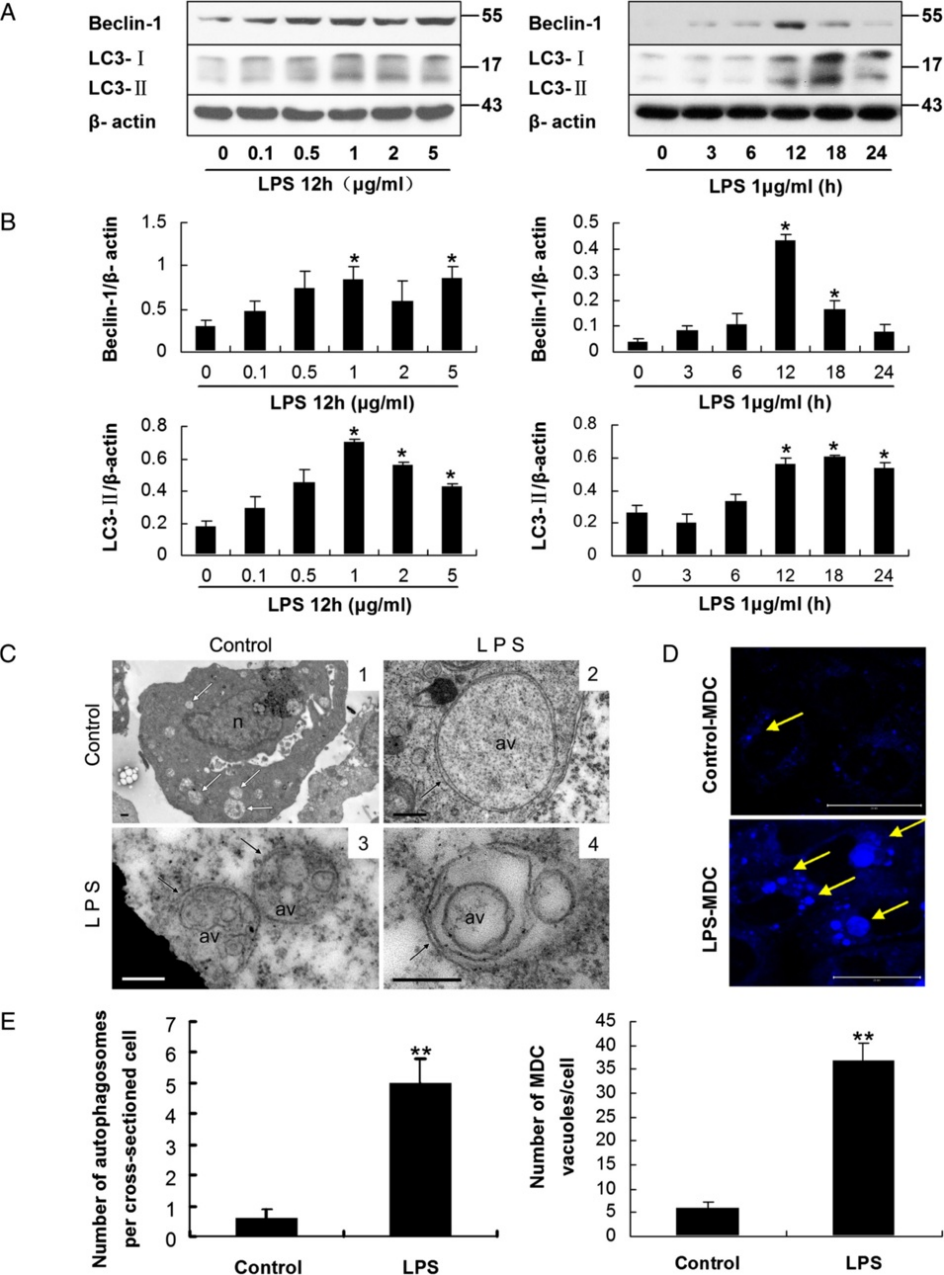
**LPS stimulation induced autophagy in HMrSV5 cells. (A)** Western blot analysis of Beclin-1 and LC3-II in HMrSV5 cells treated with LPS at various concentrations for 12 hours or 1 μg/ml LPS for the indicated time periods. β-actin was used as a loading control. **(B)** Densitometric anaysis of the blots showing the ratios of Beclin-1 and LC3-II to β-actin. **(C)** Transmission electron microscopy (TEM) of LPS-induced autophagy. Single-membrane phagosomes were seen in image 1. Image 2 shows typical double-membrane autophagosomes. Image 3 and 4 show multilayer structures. n, nucleus; av, autophagic vacuole; white arrows, single-membrane compartments; black arrows, double-membrane or multilayer structures. Scale bars: image1: 0.5 μm; image 2, 3 and 4: 200 nm**. (D)** Autophagic vacuoles were labeled with monodansylcadaverine (MDC, blue). Scale bars: 20 μm. **(E)** Graphs display quantitation of the number of autophagosomes per cross-sectioned cell (left panel) and the number of MDC-labeled autophagosomes per cell (right panel). Data are mean values ± SD (n ≥3). *p < 0.05 (vs. control); **p < 0.01 (vs. control).

Light chain 3 (LC3) exists in two forms, the 18 kDa cytosolic form (LC3-I), and the 16 kDa processed form (LC3-II) which is located on the autophagosomal membrane and a definitive marker of autophagosome formation [21]. Beclin-1, a protein factor that activates the Class III phosphoinositide 3-kinase (PI3KC3) complex [22], is another essential autophagy related protein for the eventual formation of the autophagosome [23]. Following treatment of HMrSV5 cells with LPS at concentrations of 0, 0.1, 0.5, 1.0, 2.0 and 5.0 μg/ml for 12 hours, western blotting (WB) demonstrated a dose-dependent increase in expression of Beclin-1 and LC3-II (Figure 2A and B). Apparently, after treatment with 1.0 μg/ml LPS, the amount of Beclin-1 and LC3-II in cells increased significantly (Figure 2A and B). Following treatment with 1.0 μg/ml LPS for 0, 3, 6, 12, 18 and 24 hours, respectively, the expression of Beclin-1 and LC3-II increased in a time-dependent manner with a peak at 12 hours, and then declined (Figure 2A and B). According to the results of WB and the viability assays, a concentration of 1.0 μg/ml LPS and a time point of 12 hours were chosen for further experiments.

**Figure 3:**
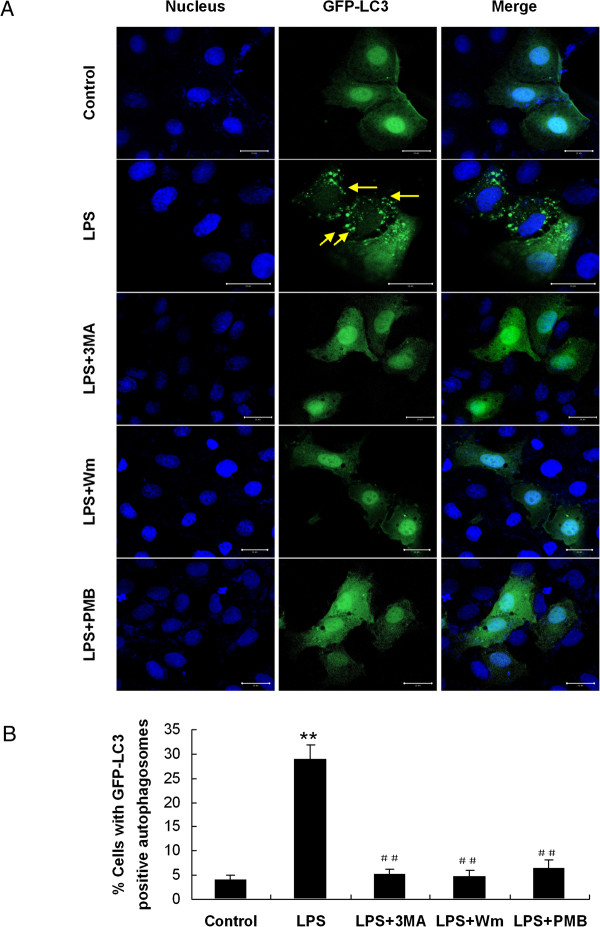
**Induction or inhibition of autophagy by pharmacological agents.** Cells transiently transfected with the GFP-LC3 plasmid were treated with combination of drugs: control, LPS (1.0 μg/ml), LPS + 3-methyladenine (3-MA, 10 mM), LPS + wortmannin (Wm, 50 nM), or LPS + Polymyxin B (PMB, 100 μg/ml). **(A)** Autophagosomes were defined as GFP-LC3 puncta. DAPI was used to label nuclei (blue). Scale bars: 20 μm. Arrows indicate punctate GFP-LC3 (green). **(B)** Graph displays the percentage of cells with GFP-LC3-positive autophagosomes. **p < 0.01 (vs. control), ##*p* < 0.01 (vs. LPS).

Autophagosome formation could be confirmed further by fluorescence microscopic analysis of GFP-LC3 cells. HMrSV5 cells were transiently transfected with plasmids encoding GFP-LC3 and then incubated with 1.0 μg/ml LPS for 12 hours. It was observed that the transiently transfected cells exhibited characteristic fluorescent punctate GFP-LC3 (LC3-II) while green fluorescence of control cells remained cytosolic and diffuse (Figure 3).

Monodansylcadaverine (MDC), a specific marker for autolysosomes [24], was also applied to confirm the induction of autophagy in treated HMrSV5 cells. As shown in Figure 2D, only basal levels of autophagy were observed in control cells, while increased number of vesicles as well as their size, which was indicated by the characteristic MDC staining, could be seen in the cells treated with LPS (Figure 2D and E, right panel).

Transmission electron microscopy (TEM) demonstrated that after exposure of LPS for 12 hours, the number of canonical double-membrane autophagosomes in HMrSV5 cells was significantly higher than that of control cells (Figure 2C and E, left panel).

### LPS-induced autophagy enhanced intracellular bactericidal activity and the co-localization of *E. coli* with autophagosomes

**Figure 4:**
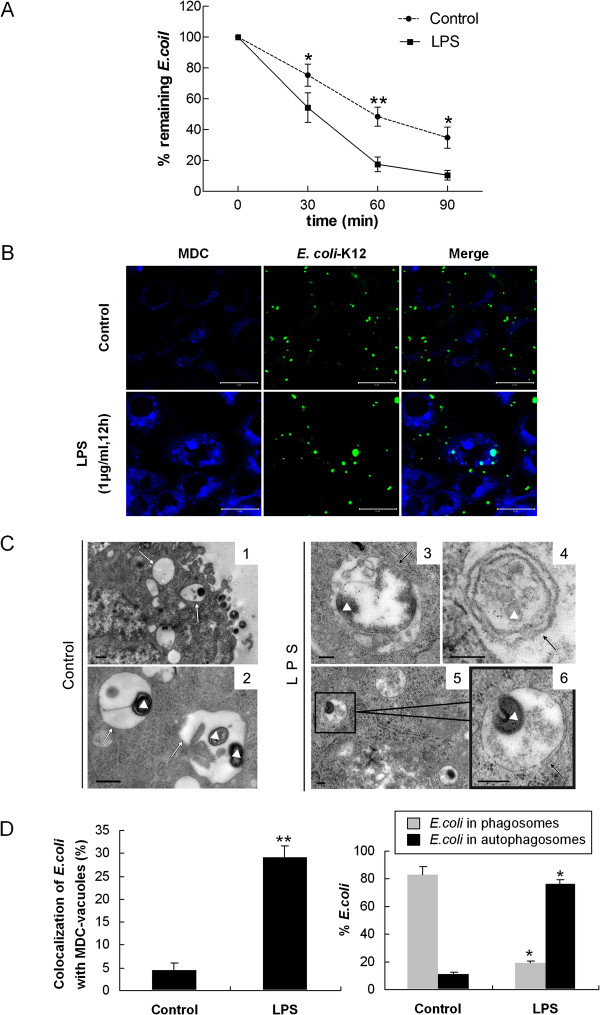
**LPS-induced autophagy promoted intracellular bactericidal activity and the co-localization of *E. coli *with autophagosomes. (A)** Bacterial killing assays for *E. coli* were performed in HMrSV5 cells treated with or without LPS (1 μg/ml, 12 hours). *E. coli* (ATCC: 25922) (MOI: 20) were incubated with the cells for 60 min (t = 0). The cells were lysed at 30, 60, 90 min later with sterile distilled water and the c.f.u. was counted. Percentage of remaining *E.coli* (%) = remaining bacteria at each time point / bacteria present at 0 min × 100. Graph represents the mean values ± SD of percentage of remaining *E.coli* at different time points from n ≥ 3 experiments. **(B)** HMrSV5 cells were infected with fluorescent *E. coli* (K-12 strain, green) for 1 hour, washed and incubated for an additional 12 hours in the presence or absence of LPS. Autophagic vacuoles were labeled with MDC (blue). Scale bars: 20 μm. **(C)** Representative TEM images of *E.coli* in autophagosomes. Images 1 and 2 show *E.coli* were engulfed in typical single-membrane phagosomes in control cells. However, more *E.coli* were harboured in double-membrane autophagosomes in LPS-treated cells (images 3–6). White triangles, *E.coli*; white arrows, single-membrane compartments; black arrows, double-membrane autophagosomes. Scale bars: image 1 and 2: 0.5 μm; image 3, 4, 5 and 6: 200 nm. **(D)** The left graph shows quantitation of the co-localization of *E. coli* with the MDC-labeled autophagosomes in Figure 4B. The right graph indicates the quantitation of 100 internalized *E. coli* per experimental condition in Figure 4C (mean values ± SD, n ≥ 3). *p < 0.05 (vs. control); **p < 0.01 (vs. control).

The effect of activation of autophagy on *E. coli* viability was monitored by the percentage of remaining *E.coli*, which was calculated by direct scoring of bacterial colony-forming units (CFU) on bacteriological media [7]. The percentage of remaining *E.coli* was 10.55 ± 3.07% in LPS pretreated cells versus 34.82 ± 6.89% in control samples after 90 min incubation (*p* < 0.05) (Figure 4A), indicating that induction of autophagic pathways by LPS in infected HMrSV5 cells could restrict the growth of *E. coli*.

To further investigate whether autophagy mediates intra-cellular antimicrobial activity in HMrSV5 cells, we analyzed the recruitment of LC3-II to *E. coli*. Following treatment with LPS, cells were infected with fluorescent *E. coli* and autophagic vacuoles were labeled with MDC. The co-localization of *E. coli* with MDC-labeled autophagic vacuoles at 1 hour post-infection in HMrSV5 cells was quantified. Compared to control cells, LPS-activated HMrSV5 cells exhibited a markedly increased rate of *E. coli* co-localization with MDC-labeled autophagic vacuoles (Figure 4B and D, left panel). As shown in Figure 4D (left panel), the rate of *E. coli* co-localization with MDC-labeled vacuoles in LPS-treated cells was 29.18 ± 2.55%, while in control cells it was 4.44 ± 1.65% (*p* < 0.01).

The effect of LPS-induced autophagy on *E. coli* limitation was also verified by electron microscopy. The TEM study showed that following stimulation of cells with LPS, 76% of *E. coli* was engulfed in double-membrane-bound autophagosomes, while in control cells, only 9% of *E. coli* was harboured in autophagosomes (Figure 4C and D, right panel). In contrast to LPS-treated cells, 83% of *E. coli* in control cells was resided in single-membrane phagosomes (Figure 4C, Figures, 1, 2 and 4D, right panel).

### Inhibition of autophagy by pharmacological inhibitors reduced LPS-induced bactericidal activity and the co-localization of *E. coli* with autophagosomes

**Figure 5:**
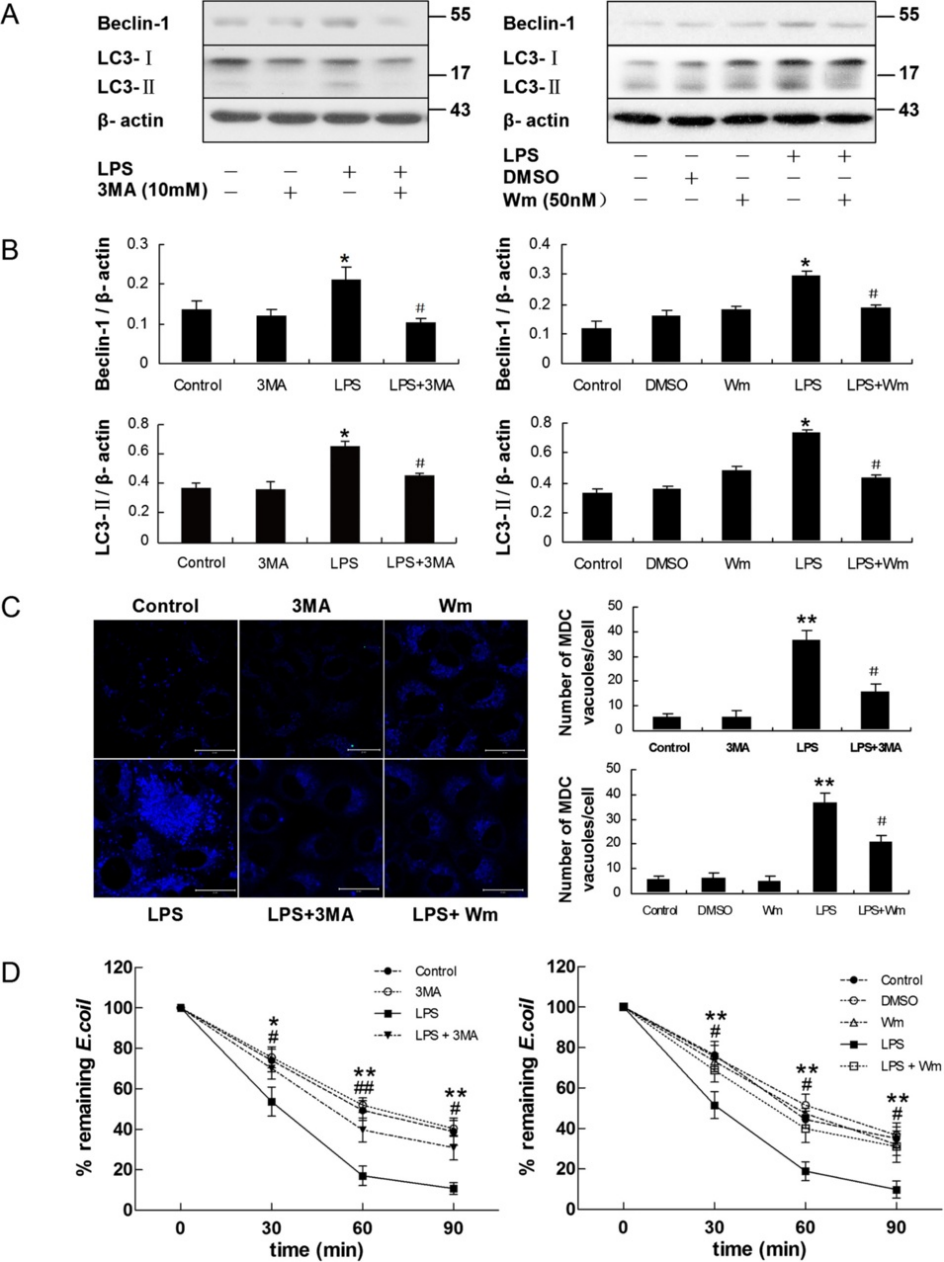
**Inhibition of autophagy by pharmacological inhibitors reduced LPS-induced bactericidal activity.** HMrSV5 cells were treated for 12 hours in the absence (control) or presence of LPS (1.0 μg/ml), DMSO, 3-MA (10 mM), Wm (50 nM), LPS + 3-MA or LPS + Wm. **(A)** The panel shows a western blot probed with antibodies against Beclin-1, LC3-II or β-actin. **(B)** Densitometric analysis of Beclin-1 or LC3-II in Figure 5A; β-actin was used as a loading control. **(C)** Autophagic vacuoles were labeled with MDC (blue) in the left panel. Scale bars: 20 μm. The graphs on the right panel represent quantitation of the number of MDC-labeled autophagosomes per cell. *p < 0.05 in Figure 5B (vs. control); ** *p* < 0.01 in Figure 5C (vs. control); # *p* <0.05 in Figure 5B and 5C (vs. LPS) **(D)** Graphs represent percentage of remaining *E.coli* in each group as described above. Data represent mean values ± SD (n ≥ 3). * and ** denote *p* < 0.05 and *p* < 0.01 respectively (LPS vs. control); # and ## denote *p* < 0.05 and *p* < 0.01 respectively (LPS + 3MA or LPS + Wm vs. LPS).

It was reported that the progression of autophagy was inhibited by the PI3K inhibitors, 3-methyladenine (3-MA) [3,7,22] and wortmannin (Wm) [7,25]. To demonstrate whether autophagy played a role in the bactericidal function of HMrSV5 cells, HMrSV5 cells were pre-incubated with 10 mM 3-MA or 50 nM Wm for 1 hour, respectively, and then treated with LPS for 12 hours. As shown in Figure 5A and B, both 3-MA and Wm pretreatment reduced the levels of Beclin-1 and LC3-II. In line with WB data, both 3-MA and Wm markedly diminished the accumulation of MDC (Figure 5C) and formation of GFP-LC3 puncta (Figure 3) in LPS-treated cells.

To further investigate the role of autophagy in limiting *E. coli* growth, we compared the growth of *E. coli* in cells with or without pharmacological inhibitors. As depicted in Figure 5D, LPS-induced bactericidal activity in HMrSV5 cells was significantly abrogated by treatment with either 3-MA or Wm.

**Figure 6:**
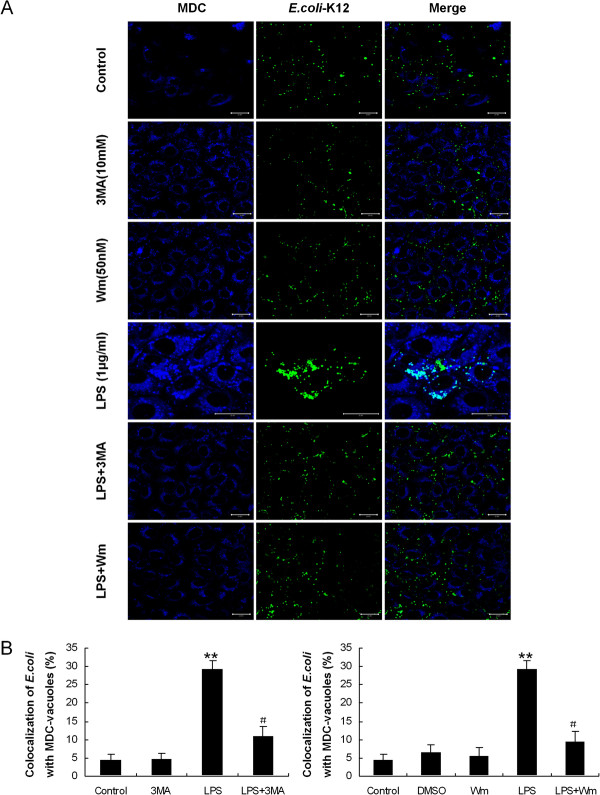
**Inhibition of autophagy by pharmacological inhibitors reduced the co-localization of *E. coli *with autophagosomes. (A)** HMrSV5 cells were infected with fluorescent *E. coli* (green) for 1 hour. Following phagocytosis, HMrSV5 cells were exposed for 12 hours in control condition, LPS (1.0 μg/ml), 3-MA (10 mM), Wm (50 nM), LPS + 3-MA or LPS + Wm. Cells were labeled with MDC (blue) for the detection of autophagic vacuoles formation. Scale bars: 20 μm. **(B)** Quantitation of the co-localization of *E. coli* with the MDC-labeled autophagosomes in Figure 6A (mean values ± SD, n ≥ 3). ** *p* < 0.01 (vs. control); # *p* < 0.05 (vs. LPS).

We analyzed the co-localization of *E. coli* with autophagosomes in HMrSV5 cells pretreated with 3-MA or Wm by confocal fluorescence microscopy. As expected, suppression of autophagy by 3-MA or Wm also attenuated the co-localization of *E. coli* with autophagosomes (Figure 6A). Following the infection, the rate of co-localization of *E. coli* with MDC-labeled autophagosomes in LPS-treated cells was approximately 29.18 ± 2.55% , while in 3-MA or Wm pretreated cells was approximately 10.95 ± 2.65% and 9.39 ± 2.78%, respectively (Figure 6B).

### Downregulation of autophagy by Beclin-1 siRNA reduced LPS-induced bactericidal activity and the co-localization of *E. coli* with autophagosomes

**Figure 7:**
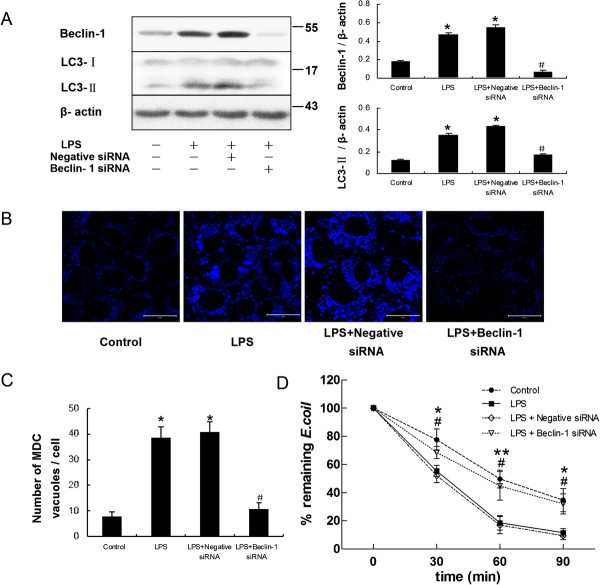
**LPS-induced bactericidal activity was attenuated after deletion of Beclin-1 by siRNA in HMrSV5 cells.** After transiently transfected with negative control siRNA or Beclin-1 siRNA, the HMrSV5 cells were incubated with LPS (1.0 μg/ml) for 12 hours. **(A)** The left panel shows representative western blots probed with antibodies against Beclin-1 and LC3-II. The right panel shows densitometric analysis of Beclin-1 and LC3-II in the left panel; β-actin was used as a loading control. **(B)** After transfection, MDC-labeled autophagic vacuoles were observed. Scale bars: 20 μm. **(C)** Quantitation of the number of MDC-labeled autophagosomes per cell in Figure 7B. * *p* < 0.05 in Figure 7A and 7C (vs. control); # *p* < 0.05 in Figure 7A and 7C (vs. LPS). **(D)** Graph represents percentage of remaining *E.coli* at different time points in each group treated as described above. Data are mean values ± SD (n ≥3). * and ** denote *p* < 0.05 and *p* < 0.01 respectively (LPS vs. control); # denote *p* < 0.05 (LPS + Beclin-1 siRNA vs. LPS).

To more specifically determine whether LPS-induced antimicrobial activity was dependent on autophagy, short interfering RNA (siRNA) specific for Beclin-1 was used to transfect the HMrSV5 cells and block autophagic responses. Figure 7A shows that knockdown of Beclin-1 effectively reduced expression of Beclin-1 and LC3-II protein. Meanwhile, fewer autophagic vacuoles labeled by MDC were observed in HMrSV5 cells transfected with Beclin-1 siRNA (Figure 7B and C).

**Figure 8:**
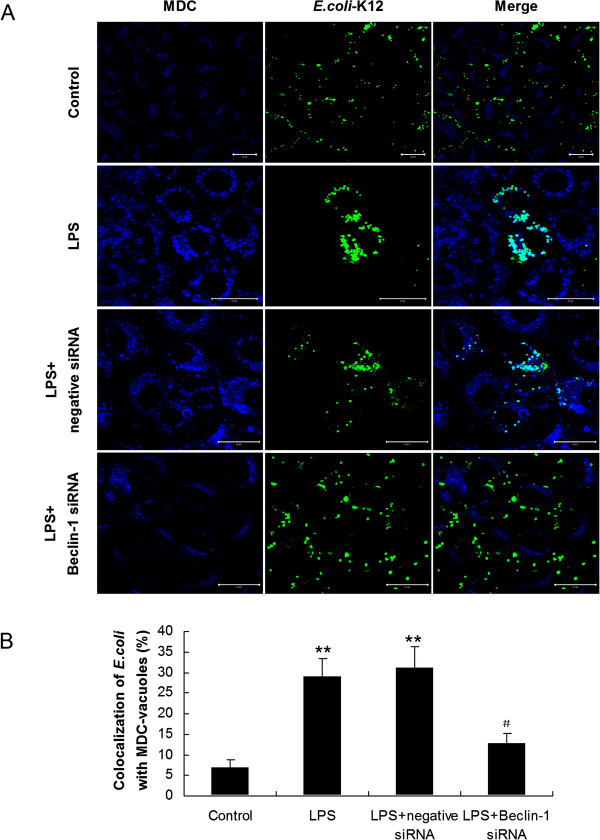
**Down regulation of Beclin-1 reduced the co-localization of *E. coli *with autophagosomes. (A)** HMrSV5 cells transfected with negative control siRNA or Beclin-1 siRNA were infected with fluorescent *E. coli* (green) for 1 hour of uptake, followed by a 12 hours chase in LPS (1.0 μg/ml). Afterwards, autophagic vacuoles were labeled with MDC (blue). Scale bars: 20 μm. **(B)** Quantitation of the co-localization of *E. coli* with the MDC-labeled autophagosomes in Figure 8A (mean values ± SD, n ≥ 3). **p < 0.01 (vs. control); # *p* < 0.05 (vs. LPS).

We subsequently examined the bactericidal activity of the siRNA-transfected cells in response to *E. coli*. Compared with control cells incubated with LPS alone, loss of Beclin-1 in HMrSV5 cells markedly attenuated bactericidal activity induced by LPS (Figure 7D). In addition, we further used MDC staining to look for *E. coli*-targeted autophagosomes. Consistent with the pharmacological inhibition of autophagy by 3-MA and Wm, co-localization of *E. coli* with MDC-labeled autophagosomes decreased from 28.98 ± 4.23% to 12.88 ± 2.34% (*p* < 0.05) upon down-regulation of the Beclin-1 gene in HMrSV5 cells (Figure 8).

### LPS induced autophagy via Toll-like receptor 4 (TLR4) dependent signaling in HMrSV5 cells

**Figure 9:**
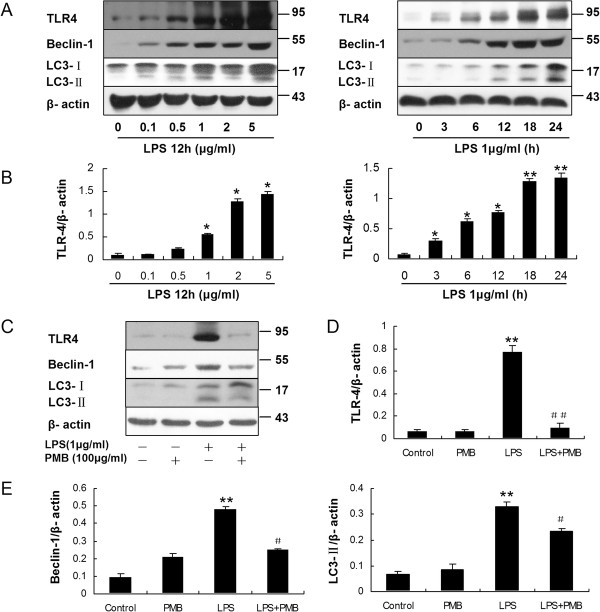
**LPS induced autophagy is dependent on TLR4 in HMrSV5 cells. (A)** Western blot analysis of TLR4, Beclin-1 and LC3-II in HMrSV5 cells treated with LPS at different concentrations for 12 hours or 1 μg/ml LPS for the indicated time periods. β-actin was used as a loading control. **(B)** Densitometric analysis of the blots showing the ratios of TLR4 to β-actin in Figure 9A. **(C)** HMrSV5 cells were stimulated for 12 hours in the absence (control) or presence of LPS (1.0 μg/ml), PMB control (100 μg/ml), LPS + PMB. The panel show western blot probed with antibodies against TLR4, Beclin-1, LC3-II or β-action. **(D and E)** Densitometric analysis of TLR4, Beclin-1 or LC3-II in Figure 9C; β-actin was used as a loading control. Data are mean values ± SD (n ≥3). * and ** denote *p* < 0.05 and *p* < 0.01 respectively (vs. control). # and ## denote *p* < 0.05 and *p* < 0.01 respectively (vs. LPS).

After incubation HMrSV5 cells with LPS, a ligand for TLR4, the expression of TLR4 increased in a dose-dependent and time-dependent way, as determined by WB (Figure 9A and B). Interestingly, TLR4 protein increased quickly at early stage (3 ~ 6 hours), which was earlier than the increase of LC3-II protein. It was also observed that expression levels of both Beclin-1 and LC3-II protein were significantly diminished in cells pretreated with 100 μg/ml Polymyxin B (PMB) (Figure 9C, D and E), an antibiotic binding to lipid A, which is the component of LPS responsible for receptor binding and cellular signaling [10]. Moreover, PMB pretreatment decreased GFP–LC3 aggregation as demonstrated by immunofluorescent microscopy (Figure 3).

**Figure 10:**
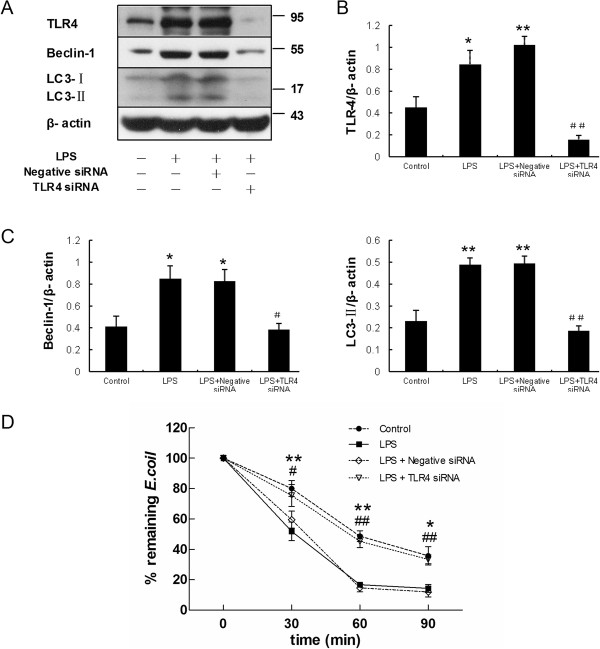
**Knockdown of TLR4 inhibits LPS induced autophagy and bactericidal activity.** After transiently transfected with negative control siRNA or TLR4 siRNA, the HMrSV5 cells were incubated with LPS (1.0 μg/ml) for 12 hours. **(A)** The panel shows representative images of western blots probed with antibodies against TLR4, Beclin-1, LC3-II and β-actin. **(B)** Densitometric analysis of the blots showing the ratios of TLR4 to β-actin in Figure 10A. **(C)** Densitometric anaysis of the blots showing the ratios of Beclin-1 and LC3-II to β-actin in Figure 10A. * and ** denote *p* < 0.05 and *p* < 0.01 respectively in Figure 10B and 10C (vs. control); # and ## denote *p* < 0.05 and *p* < 0.01 respectively in Figure 10B and 10C (vs. LPS). **(D)** Graph represents percentage of remaining *E.coli* at different time points in each group treated as described above. Data are mean values ± SD (n ≥3). * and ** denote *p* < 0.05 and *p* < 0.01 respectively (LPS vs. control); # and ## denote *p* < 0.05 and *p* < 0.01 respectively (LPS + TLR4 siRNA vs. LPS).

In addition, knockdown of TLR4 with TLR4 siRNA markedly decreased expression of Beclin-1 and LC3-II protein activated by LPS incubation (Figure 10A, B and C), which indicated that loss of TLR4 attenuated LPS-induced autophagy. Furthermore, as shown in Figure 10D, TLR4 siRNA impaired intracellular bactericidal activity induced by LPS.

## Discussion

Although aberrant autophagy is observed in many bacterial infectious diseases, the role of autophagy in PD-related peritonitis remains unknown. Our study has investigated the role of autophagy in PMCs against intracellular *E.coli.* We demonstrated that LPS could induce autophagy in HMrSV5 cells. LPS enhanced the intracellular bactericidal activity of HMrSV5 cells and promoted the co-localization of *E.coli* (K12-strain) with autophagosomes. Moreover, treatment with microtubule-disrupting agents such as 3-MA or Wm or Beclin-1 siRNA, markedly attenuated the intracellular bactericidal activity of HMrSV5 cells and the co-localization of *E. coli* with autophagosomes induced by LPS treatment. Furthermore, knockdown of TLR4 vanished LPS-induced autophagy and bactericidal activity. These data collectively suggest that autophagy activated by LPS via TLR4 represents an innate defense mechanism for inhibiting intracellular *E. coli* replication.

Autophagy is a process traditionally known to contribute to cellular cleaning via the removal of intracellular components in lysosomes [26]. Recently, our colleagues reported that LPS stimulation led to autophagy in cultured peritoneal mesothelial cells [27]. In keeping with their reports, our data revealed that LPS induced accumulation of LC3-II in a time- and dose-dependent manner in HMrSV5 cells, as indicated by an increased aggregation of GFP-LC3 puncta and a higher number of autophagosome-like MDC-labeled vacuoles. Furthermore, HMrSV5 cells pretreated with 3-MA, Wm or Beclin-1 siRNA displayed defective autophagy induction in response to LPS. These results indicate that LPS is a general stimulant of autophagic activity in PMCs. In addition, our study showed the viability of LPS-treated cells had no significant difference compared to the control group. It has been demonstrated that exposure of PMCs to LPS resulted first in autophagy and later, apoptosis [27]. Apoptosis was only observed under higher concentrations of LPS (5 to 10 μg/ml) exposure for 48 hours in HMrSV5 cells [27]. We could not detect apoptosis in HMrSV5 cells following the incubation with lower doses of LPS (0-5 μg/ml) for shorter time periods (0-24 h) in present study, which was consistent with the previous report [27]. These observations indicated that incubation of 1 μg/ml LPS for 24 hours was sufficient to induce autophagy but not apoptosis in HMrSV5 cells.

During infection, the ability of macroautophagy to remove large cytoplasmic structures with selectivity enables this pathway to be used to clear intracellular bacteria, parasites, and viruses (i.e., xenophagy) [1,8,9]. Several medically important human pathogens are degraded in vitro by xenophagy, including bacteria (e.g., *group A streptococcus*, *Mycobacterium tuberculosis*, *Shigella flexneri*, *Salmonella enterica*, *Listeria monocytogenes*, and *Francisella tularensis*), viruses such as *herpes simplex virus type 1* (*HSV-1*) and *chikungunya virus*, and parasites such as *Toxoplasma gondii*[9]. We therefore wondered whether induction of autophagy could affect the growth of *E. coli* in infected HMrSV5 cells. We found that stimulation of autophagy by LPS in infected HMrSV5 cells could lead to degradation of *E. coli* within autophagosomes. Furthermore, we observed that 3-MA or Wm blockade of autophagy markedly attenuated the co-localization of *E. coli* with autophagosomes, leading to a defect in bactericidal activity. To more specifically determine whether autophagy affect the elimination of *E.coli*, Beclin-1 siRNA was employed to inhibit autophagy. As expected, fewer *E.coli* were targeted to the autophagosomes, and consequently more remaining *E.coli* were observed in cells deficient in Beclin-1. Taken together, these data demonstrated that the effect of LPS on bactericidal activity was dependent on the induction of autophagy.

LPS is the ligand for TLR4, and it also exerts multiple cellular effects by inducing signaling through TLR4 [10]. The activation of TLR4 by LPS in peritoneal mesothelial cells might result in a massive influx of leukocytes in the peritoneal cavity, leading to the development of peritoneal dysfunction or peritoneal fibrosis [28]. It was demonstrated that TLR4 served as a previously unrecognized environmental sensor for autophagy [10]. Therefore we further investigated whether TLR4 played roles in LPS-induced autophagy in HMrSV5 cells. Our results showed that the LPS treatment increased the expression of TLR4 protein significantly in a dose-dependent and time-dependent way. Moreover, the increased expression of TLR4 protein occurred earlier than the increase of LC3-II protein. Pretreated with PMB, a TLR4 inhibitor, displayed defective autophagy activation as indicated by the significantly decreased expression of both Beclin-1 and LC3-II protein as well as the decreased GFP–LC3 aggregation in cells. Consistent with the pharmacological inhibition of TLR4, knockdown of TLR4 with TLR4 siRNA also led to reduction of autophagy-associated proteins. Importantly, LPS-induced bactericidal activity in HMrSV5 cells was significantly decreased after knockdown of TLR4. To sum up, these results demonstrated that upregulation of autophagic response by LPS was dependent on TLR4 signaling in HMrSV5 cells.

## Conclusion

The present data revealed that LPS-induced autophagy in HMrSV5 cells enhances both the co-localization of *E. coli* with autophagosomes and intracellular bactericidal activity. The upregulation of autophagic response induced by LPS was dependent on the activation of TLR4 signaling. These results indicate that LPS-induced autophagy is at least partially responsible for the growth restriction of *E. coli* in PMCs. Developing strategies of selectively stimulating autophagy in infected cells may be considered as a new method for dealing with hard-to-eliminate *E. coli.* Further and precise in vivo studies may shed light on how autophagy combats invasive pathogens inside the host cells.

## Competing interests

The authors declare that they have no competing interests.

## Authors’ contributions

XY conceived of the study, participated in its design and coordination and helped to draft the manuscript. JWang performed most of the experiments, analyzed data and wrote the manuscript. XRF and YJZ participated in western blotting, cell viability assay and helped to perform the statistical analysis. JJF participated in immunofluorescence assays. JWu participated in cell culture. XHL and RH participated in transfection and bacterial killing assay. ZJL and FXH participated in checking and analyzing data. XQY participated in its design and modified the the manuscript. All authors have read and approved the final manuscript.
